# Enhanced Hand Gesture Recognition with Surface Electromyogram and Machine Learning

**DOI:** 10.3390/s24165231

**Published:** 2024-08-13

**Authors:** Mujeeb Rahman Kanhira Kadavath, Mohamed Nasor, Ahmed Imran

**Affiliations:** College of Engineering and Information Technology, Ajman University, Ajman P.O. Box 346, United Arab Emirates; m.nasor@ajman.ac.ae (M.N.); a.imran@ajman.ac.ae (A.I.)

**Keywords:** electromyogram, EMG, Myo armband, EMG sensor, hand gestures, cross-validation, machine learning, random forest, AUC-ROC

## Abstract

This study delves into decoding hand gestures using surface electromyography (EMG) signals collected via a precision Myo-armband sensor, leveraging machine learning algorithms. The research entails rigorous data preprocessing to extract features and labels from raw EMG data. Following partitioning into training and testing sets, four traditional machine learning models are scrutinized for their efficacy in classifying finger movements across seven distinct gestures. The analysis includes meticulous parameter optimization and five-fold cross-validation to evaluate model performance. Among the models assessed, the Random Forest emerges as the top performer, consistently delivering superior precision, recall, and F1-score values across gesture classes, with ROC-AUC scores surpassing 99%. These findings underscore the Random Forest model as the optimal classifier for our EMG dataset, promising significant advancements in healthcare rehabilitation engineering and enhancing human–computer interaction technologies.

## 1. Introduction

**Figure 1:**
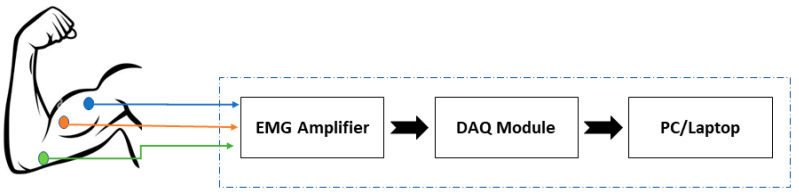
Single-channel EMG recorded using surface electrodes.

**Figure 2:**
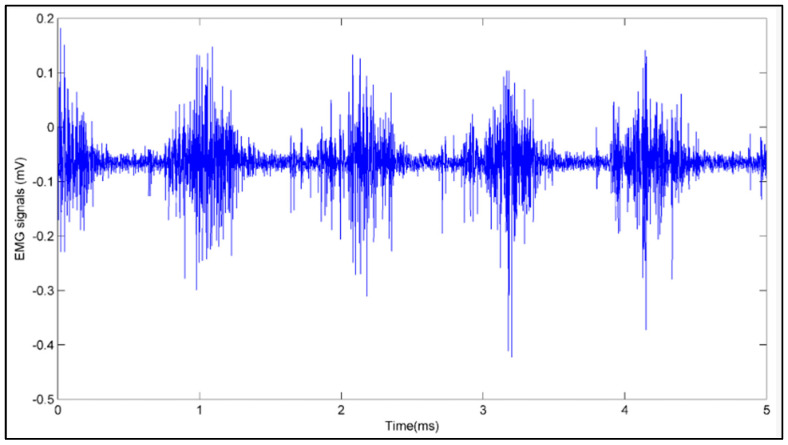
Single-channel EMG signal recorded from the biceps.

Electromyography (EMG) is a fascinating field of study that explores the electrical activity of muscles and nerves in the body. Using EMG, one can record the electrical impulses generated by muscle fibers, which reveal important details about the condition and operation of the muscular system. It can offer a dependable method of detecting and classifying muscle activity associated with various hand movements [1,2]. EMG is a diverse field with origins in engineering and neuroscience, presenting a variety of potential for research and innovation. For example, EMG can be used to control prosthetic limbs. By detecting the electrical signals generated by residual muscle activity in an amputee’s stump, EMG can be used to manage a prosthetic limb’s motion [3]. This technique, known as myoelectric control, has shown promising results in clinical trials and is becoming increasingly popular in the field of prosthetics [4]. One widely used method for acquiring EMG signals involves placing EMG electrodes around specific muscle sites and using an EMG amplifier module for signal acquisition. This method is non-invasive, ensuring no harm to the body [5,6]. Figure 1 illustrates a single-channel EMG recording setup. It entails attaching two main electrodes (also called active electrodes) and a common electrode (also known as a reference electrode) to the subject’s biceps muscle. Figure 2 displays the matching single-channel EMG signal, where each burst represents a voluntary muscle contraction.

**Figure 3:**
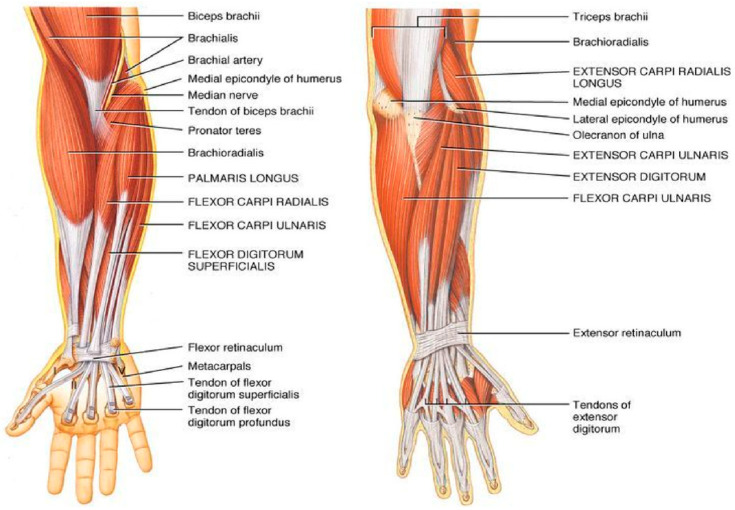
Flexor and extensor muscles responsible for hand gestures.

The dexterity of our finger movements relies on the intricate coordination of various muscles. The flexor digitorum profundus and flexor digitorum superficialis muscles facilitate the flexing of our fingers, while the extensor digitorum communis and extensor indicis muscles are responsible for straightening them [7,8]. This coordination is illustrated in Figure 3.

Through the analysis of EMG data, which captures the electrical impulses sent by these muscles, we can unravel the complex choreography that forms the basis of every movement of the fingers. Pinpointing a single muscle maestro for each finger movement remains a challenge with EMG. However, the electrical signals captured by EMG electrodes, strategically placed around the forearm, reveal characteristic shifts that can tell us which fingers are playing a role in the motion [9]. Beyond deciphering finger movements, EMG unlocks a world of possibilities. For those with prosthetics, EMG from remaining muscles translates into natural control of grip strength and finger movements [10]. Assistive technology benefits too, with EMG empowering individuals to operate wheelchairs, robotic arms, and communication tools [11]. Human–computer interaction receives a makeover with EMG-controlled cursors, games, and even gesture-based commands [12]. Rehabilitation sees EMG used to monitor muscle activity during exercises, optimizing treatment plans [12,13]. Virtual reality receives a touch upgrade, as EMG allows for more realistic manipulation of virtual objects [14]. The potential extends further, with EMG facilitating gesture recognition for smart home control, menu navigation, and even playing musical instruments through specific hand movements [15].

Recognizing gestures from arm EMG signals necessitates advanced signal processing and analysis techniques, such as pattern recognition and feature extraction. Machine learning algorithms are particularly adept at classifying signals or images based on the extracted features. By harnessing the robust capabilities of ML, we can train algorithms to identify patterns within EMG data. This training enables the algorithms to accurately predict the class of unknown EMG signals, thereby effectively recognizing gestures [14].

**Figure 4:**
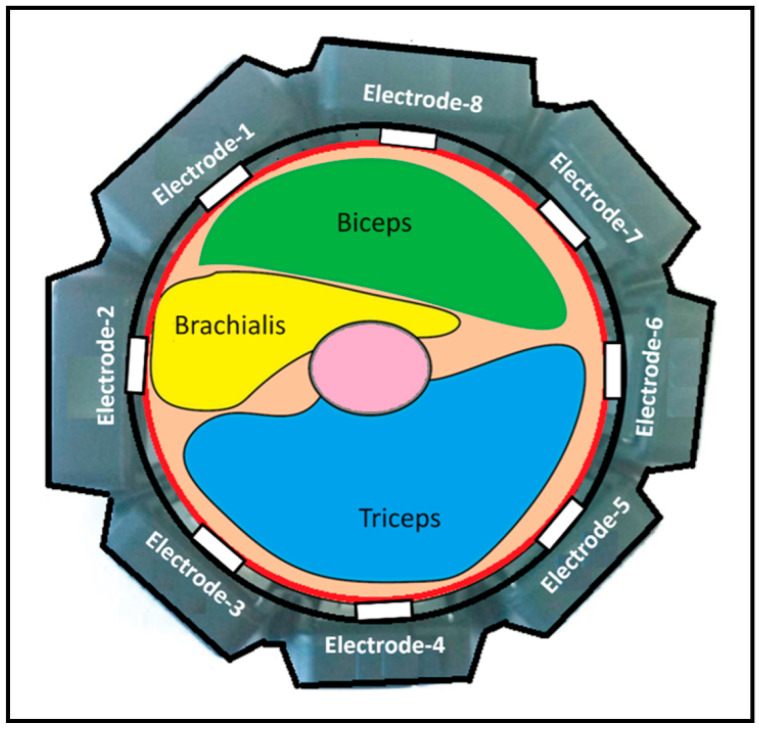
Myo armband sensor module.

**Figure 5:**
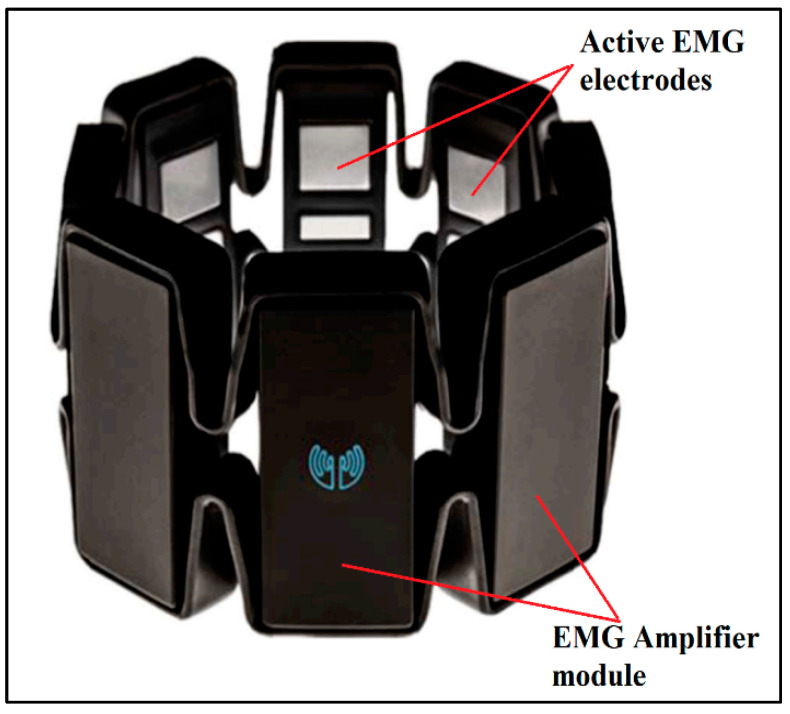
Mounting of Myo armband.

We adopted a wearable EMG sensor module called the Myo armband to record the electrical activity of the forearm muscles. The Myo armband operates by detecting and measuring the electrical activity generated by muscle contractions. As shown in Figure 4, it consists of eight electrodes strategically placed around the circumference of the forearm to capture the electrical signals produced by the muscles. When a muscle contracts, it generates an electrical signal known as an electromyogram (EMG). Figure 5 demonstrates the mounting of the Myo armband sensor on the subject’s arm for experimentation. The electrodes detect these signals by measuring the voltage differences on the skin surface caused by the underlying muscle activity.

The raw EMG signals are typically very weak, so they need to be amplified. The Myo armband contains built-in amplifiers to boost the signal strength, making it suitable for further processing. The amplified EMG signals are then filtered to remove noise and other artifacts, ensuring that the signals are clean and accurately represent muscle activity. These filtered analog signals are converted into digital signals using an analog-to-digital converter (ADC), necessary for processing by digital systems and algorithms.

Key quantities related to muscle activity include amplitude, which reflects the level of muscle activation; frequency, which provides information about the type of muscle fibers being activated and the nature of the muscle contraction; duration, which corresponds to the length of time the muscle is active during a contraction; and timing, indicating when a muscle starts and stops contracting. Additionally, analyzing changes in frequency and amplitude over time can assess muscle fatigue. These quantities are crucial for understanding muscle behavior.

The Myo armband provides eight channels of raw EMG data, which are kept separate and never mixed. We divided the raw EMG signals into 6822 segments, each 150 samples long. From these segments, ten features were extracted and combined to create the data required for building the model. The Myo armband connects wirelessly to devices using Bluetooth Low Energy (BLE) to transmit acquired data, ensuring efficient use of its battery life.

The user-friendly design of the Myo armband, lauded for its simplicity, lightness, and comfort, has made it a popular choice for researchers exploring sign language recognition in various applications like gaming and virtual reality [15]. One such study by Abreu et al. focused on Brazilian Sign Language (LIBRAS) recognition using a Myo armband and an SVM classifier [16]. Their dataset encompassed 20 static postures representing letters from the LIBRAS alphabet. However, the model achieved higher accuracy with simpler letter shapes (L: 77%, R: 91%, S: 46%, W: 95%) compared to more complex ones (F: 8%, M: 8%, P: 8%, T: 4%, U: 5%). This highlights the challenge of differentiating intricate hand formations based on EMG signals. Additionally, letters like A and S, or G and Q, despite being visually distinct, can cause misclassification due to similar signal patterns or applying pressure to the same finger. The study underscores the importance of precise gesture replication during training, emphasizing location and exerted force for the model to learn accurate representations of each sign and achieve better real-time recognition.

Mani et al. conducted a study to assess the feasibility of using the wearable Myo armband device for exploring patterns and relationships in finger movements during musical instrument playing using unsupervised learning [17]. They employed the Myo armband to aid in learning the electric keyboard, tracking finger movements and detecting muscle signals to identify played musical themes based on finger presses. Electromyography (EMG) signals from all eight channels of the Myo armband were recorded. Eleven features were extracted and processed using Linear Discriminant Analysis (LDA) for dimension reduction. Subsequently, the data were input into a Random Forest (RF) classifier. The study included EMG data collection in both single-user and multi-user scenarios, covering six distinct targets. Notably, electrodes placed on the anterior side of the device proved highly sensitive and beneficial for classifying finger movements. Findings showed that for multiple-finger chord playing on the keyboard, using two electrodes of the EMG signal achieved 95.83% accuracy. Additionally, for classifying individual musical notes, the Myo armband with its four electrodes achieved close to 75% accuracy.

Anil et al. conducted a research study utilizing machine learning with EMG signals captured by the Myo armband sensor module [18]. They applied discrete wavelet transform (DWT) to process these dynamic EMG signals, focusing on recognizing five specific hand gestures: Rest, Fist, Wavein, Waveout, and Fingerspread. Their approach achieved an impressive accuracy of 83% using a dataset of 335 samples, with 80% used for training and the remaining 20% for testing. Opting for SVM due to its effectiveness with smaller datasets, the authors employed SVM’s hyperplane to classify and separate data points. The study underscored the efficacy of DWT in effectively processing dynamic EMG signals. Overall, their research demonstrated robust accuracy in identifying various gestures across the dataset.

In another study focused on classifying EMG signals captured from the Myo armband, researchers aimed to discern specific finger activities like thumb, index, ring, and little finger movements, as well as resting states. Unlike traditional methods, Srinivasan et al., 2018 [19], explored deep learning techniques, specifically convolutional neural networks (CNNs). They implemented a model incorporating Inception Net v1 using surface electrodes. Training with 80% of the data and testing with the remaining 20%, their deep learning approach achieved an accuracy of 72.5% for classifying five finger movements, marking a significant 16.5% improvement over the commonly used SVM method. Subsequent advancements included expanding their dataset to further enhance classification capabilities.

The literature highlights challenges in using the Myo armband for gesture recognition, including dataset size, feature selection, and machine learning algorithms. Future studies should focus on increasing dataset sizes, incorporating dynamic gestures, exploring advanced machine learning algorithms, and ensuring thorough user calibration. The primary goal of this project was to identify the best-performing ML classifier for seven distinct hand gestures using EMG signals recorded by the Myo armband.

## 2. Materials and Methods

**Figure 6:**
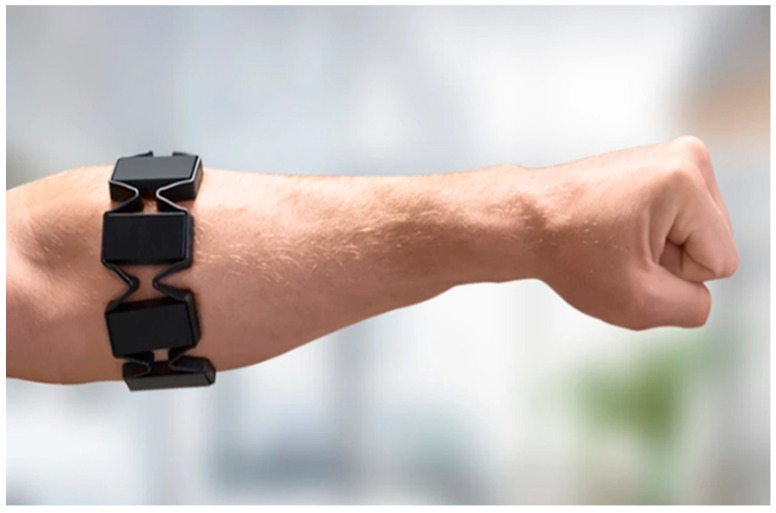
Myo armband fitted on the arm for data acquisition.

**Figure 7:**
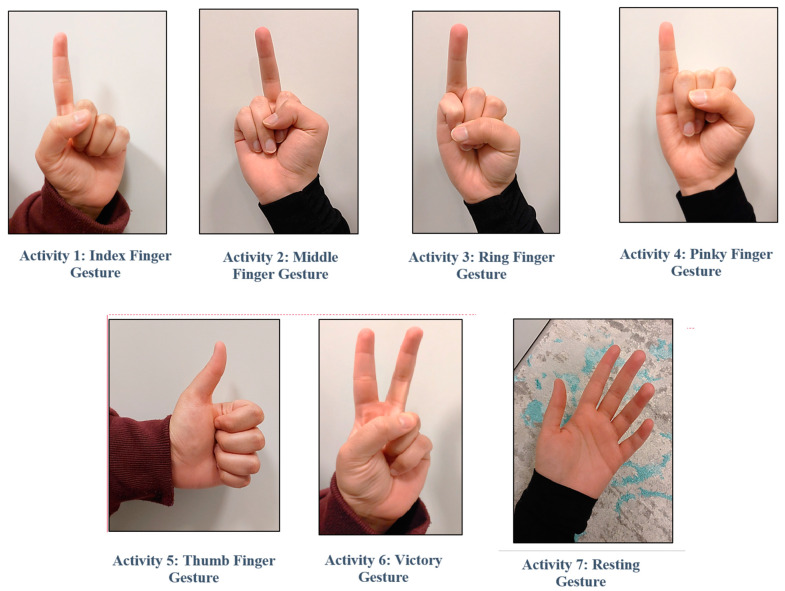
Seven hand gestures used in the study.

This study involved utilizing the Myo armband to capture EMG data associated with seven distinct hand gestures. The Myo armband was comfortably worn on the participant’s arm to capture the EMG signals, as illustrated in Figure 6. The selected hand gestures for analysis encompassed the index finger, middle finger, ring finger, pinky finger, thumb, resting gesture, and victory gesture, as visualized in Figure 7. EMG data were collected while participants performed these gestures in a predefined sequence, facilitated by a visual cue for each gesture. In this dataset, each finger movement requires nearly 1 s and is reflected as a sudden change in the EMG amplitude. Creating the dataset for this project took 18.7 min per subject, resulting in a total sample length of 223,793 at a sampling rate of 200 Hz. Finger motions typically appear near the center of each segment. We segmented the EMG data according to the hand gestures, with each segment initially consisting of 200 samples. However, the ends of each peak are considered static and do not play a significant role, so we retained 150 samples per segment. The resulting dataset contains 6822 segments, encompassing 7 classes of hand gestures. The entire trial lasted 18.7 min. For detailed information on each hand motion, including their frequencies and percentage distributions, refer to Table 1. The Myo armband collects eight-channel EMG data from the participant at a sample rate of 200 Hz.

A segment represents EMG data from one of the channels, consisting of 150 sample points, which corresponds to a specific finger motion. For each finger motion, we can have eight segments, each originating from one of the eight channels.

**Figure 8:**
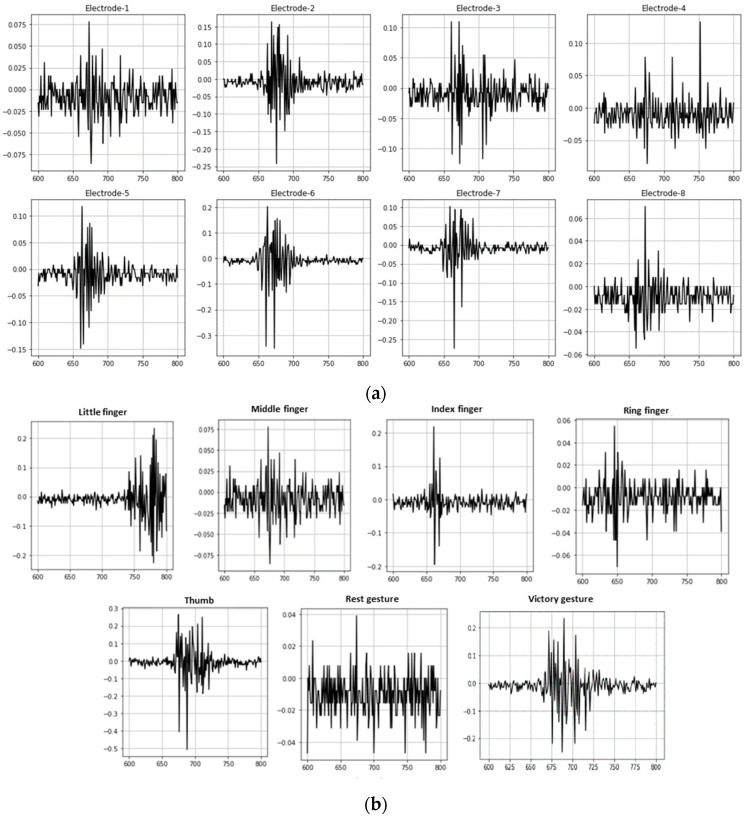
(**a**) Eight-channel (Electrode-1 to Electrode-8) raw EMG data used for the study corresponds to the index finger gesture. (**b**) Raw EMG data recorded by electrode 1 corresponds to the seven hand gestures.

The unprocessed EMG raw data corresponding to the index finger, captured over a randomly chosen segment of 200 samples (for the interval 600 to 800), are shown in Figure 8a,b, which shows the raw EMG data recorded by electrode 1, corresponding to the seven hand gestures.

**Figure 9:**
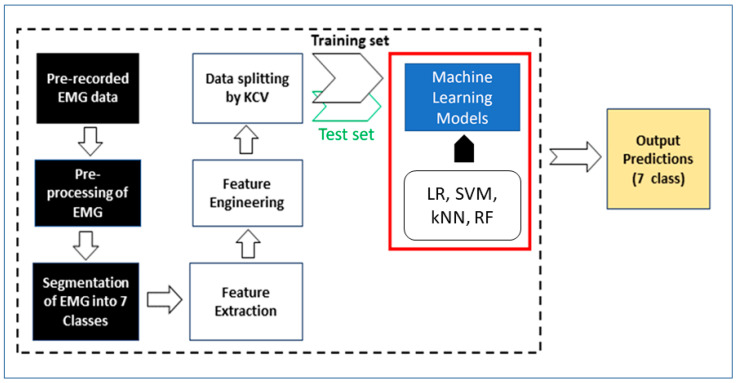
Architecture of the proposed methodology.

The architecture of the proposed methodology is presented in Figure 9. The input to the model is the raw EMG data, which then undergo a series of preprocessing steps. These steps include the application of band-pass filters (band width: 20 to 100 Hz) to remove noise and isolate relevant signal frequencies, normalization to standardize the data, rectification to convert all values to positive, and envelope extraction to capture the amplitude variations of the rectified signal. Additionally, the EMG data are segmented into seven classes based on the labels provided in the dataset.

Following these preprocessing and segmentation steps, the data are prepared for feature extraction and subsequent analysis within the model. In the subsequent stage, the algorithm extracts a diverse set of features from each segment of the EMG data. Our experiments covered statistical, time-domain, and frequency-domain features, leading us to identify the most effective ones, as described in the following section [20,21,22].

(a) Minimum (min): The smallest value in the signal over a given window.
  For a given EMG signal x(t) over a specific timeframe [t1,t2], the minimum value of the signal over the given window [t1,t2] is defined as:
  (1) $$Min\left(x\right)={min}_{t\in \left[t1,t2\right]}\left\{x\left(t\right)\right\}\;\;\;\;\;$$
(b) Maximum (max): The largest value in the signal over a given window.
  These two features together define the overall range of muscle activation within a specific timeframe.
  (2) $$Max\left(x\right)={max}_{t\in \left[t1,t2\right]}\left\{x\left(t\right)\right\}\;\;\;\;\;\;\;$$
(c) Standard Deviation (SD): Measures the amount of variation or dispersion of a set of values. Captures how spread out the EMG signal is from its mean value. A high SD indicates high variability, suggesting a mix of muscle activation levels.
  (3) $$SD\left(x\right)=\sqrt{\frac{1}{N-1}\sum_{i=1}^{N}{x(t_{i}-\mu )}^{2}}\;\;$$
  where *N* is the number of samples in the timeframe and *μ* is the mean of the signal over the window:
  (4) $$\mu \left(x\right)=\frac{1}{N}\sum_{i=1}^{N}x(t_{i})\;$$
(d) Zero Crossings (ZC): Counts the number of times the signal crosses zero, reflecting the frequency of muscle fiber recruitment and relaxation cycles. To accurately capture all zero crossings, we analyze the EMG data before the rectification stage
  (5) $$ZC\left(x\right)=\sum_{i=1}^{N}1\;[(x(t_{i}).\;x\left(t_{i+1}\right)<0)]$$
  where 1 is the indicator function, which equals 1 when the condition inside is true and 0 otherwise.
(e) Root Mean Square (RMS): The square root of the mean of the squares of the values. It represents the overall power of the signal, reflecting the average muscle activity level.
  (6) $$RMS\left(x\right)=\sqrt{\frac{1}{N}\sum_{i=1}^{N}{x(t_{i})}^{2}}\;$$
(f) Amplitude Change (AAC): Measures the change in amplitude between consecutive samples. It is useful for identifying rapid changes in muscle activity.
  (7) $$AAC\left(x\right)=\frac{1}{N-1}\sum_{i=1}^{N}\left|x\left(t_{i+1}\right)-x\left(t_{i}\right)\right|\;\;\;\;$$
(g) Amplitude of First Burst (AFB): Identifies the peak value within a specific time window after the signal crosses a threshold, indicating the initial burst of muscle activation.
  The first crossing time ($t_{c}$) of the threshold *T* is given by:
  (8) $$t_{c}=\min\left\{t\epsilon \left[t1,t2\right]\;\;\right|x\left(t\right)\geq \;T\;\;$$
  Amplitude of max burst within the window $\left[t_{c},\;t_{c}+t_{b}\right]$ can be estimated by using
  (9) $$AFB\left(x\right)={max}_{t\in \left[t_{c},\;{\;t}_{c}+t_{b}\right]\;}\;\{x\left(t\right)\}$$
(h) Mean Absolute Value (MAV): The average of the absolute values of the signal over a specified time window. Similar to RMS, it reflects the average magnitude of the signal, regardless of positive or negative values.
  (10) $$MAV\left(x\right)=\frac{1}{N}\sum_{i=1}^{N}\left|x(t_{i})\right|\;\;\;$$
(i) Length of Waveform (Len): Duration or length of the signal.
(j) Willison Amplitude (WAMP): Counts the number of times the EMG signal crosses a specific threshold within a particular time window. It provides a rough estimate of the dominant frequency of the EMG signal, potentially reflecting muscle fiber recruitment patterns.
  (11) $$\sum_{i=1}^{N}1\;[\left|(x\left(t_{i+1}\right)-x\left(t_{i}\right)\right|\geq T)]$$
  where 1 is the indicator function, which equals 1 when the condition inside is true and 0 otherwise.

In the next step, we employed feature engineering to enhance the performance of our machine learning models by selecting, transforming, and creating features. This process involved various techniques to make raw data more suitable for predictive modeling, thereby improving model accuracy and efficiency. Key techniques included identifying and selecting the most relevant features and transforming features through normalization (scaling features to a specific range) and standardization (scaling features to have zero mean and unit variance). Additionally, we applied encoding methods such as one-hot encoding, label encoding, and target encoding to convert categorical variables into numerical formats.

**Figure 10:**
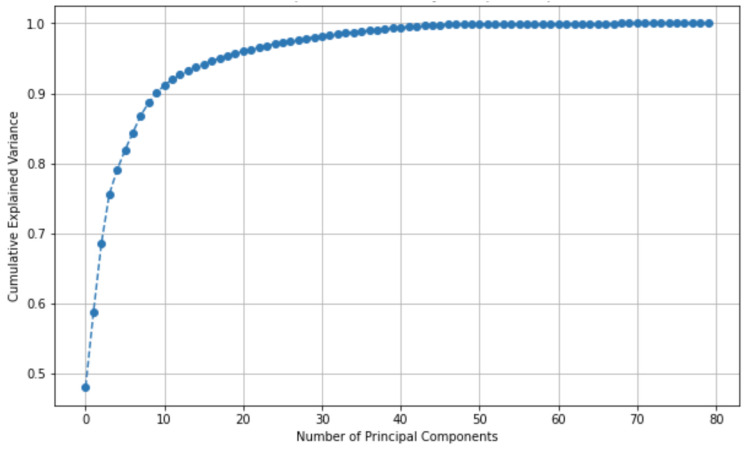
Plot of cumulative explained variance by PCA.

Initially, we built the model using all ten features from each of the eight electrodes (80 features in total). We applied z-score normalization to these features and used one-hot encoding for the seven target columns. To assess feature redundancy, we calculated the correlation coefficients between the features and found that 30 features exhibited correlations of 90% or higher. To streamline the feature set, we used Principal Component Analysis (PCA), which showed that the first 49 principal components retained 99.9% of the information, as illustrated in the cumulative PCA plot in Figure 10. Consequently, we trained the model using these 49 components, achieving nearly the same (slightly less) performance scores as in the previous stage. This comprehensive feature engineering process optimized our dataset for the subsequent machine learning tasks.

We included four widely recognized machine learning algorithms to construct our proposed hand gesture classifier: Logistic Regression (LR), Support Vector Machine (SVM), K-Nearest Neighbors (kNN), and Random Forest (RF).

LR is a linear model that predicts the probability of a binary outcome using a logistic function. It is known for its simplicity, interpretability, and efficiency in linearly separable datasets [23].

SVM is a powerful classification technique that finds an optimal hyperplane to separate data into different classes. It is effective in high-dimensional spaces and can handle non-linear relationships using kernel functions. SVM with a linear kernel finds an optimal hyperplane to separate data into different classes in a linearly separable feature space. It is efficient and works well when the classes are linearly separable. SVM with a Radial Basis function (RBF kernel) can handle non-linear relationships by mapping input data into a higher-dimensional space. It computes similarity to nearby points and is effective in capturing complex decision boundaries [24,25].

kNN is a non-parametric method that classifies new instances based on their similarity to training examples in the feature space. It is intuitive and robust but can be computationally expensive with large datasets [26].

RF is an ensemble learning method that constructs multiple decision trees during training and outputs the mode of the classes as the prediction (classification) or the average prediction (regression) of the individual trees. It is known for its robustness against overfitting and ability to handle complex interactions in data [27].

The metrics used to assess the performance of the candidate algorithms were precision, recall, F1-score, accuracy, and area under the ROC curve (AUC).

Precision is a metric that measures the accuracy of the positive predictions made by a machine learning model. It is defined as the ratio of true positive predictions to the total number of positive predictions. Precision is particularly important in situations where the cost of false positives is high [28].

(12) $$Precision=\frac{True\;Positives\;}{Total\;number\;of\;positives}$$

Recall, also known as sensitivity or true positive rate, measures the ability of a model to identify all relevant instances. It is the ratio of true positive predictions to the total number of actual positive instances. Recall is crucial when the cost of false negatives is high, such as in medical diagnoses [28].

(13) $$Recall=\frac{True\;Positives\;}{True\;Positives+False\;negatives}$$

The F1-score is the harmonic mean of precision and recall, providing a single metric that balances both concerns. It is useful when you need a balance between precision and recall and when dealing with imbalanced datasets. The F1-score ranges from 0 to 1, with 1 being the best possible score [28].

(14) $$F1=2\times \frac{Precision\times Recall\;}{TPrecision+Recall}$$

Accuracy is the ratio of correctly predicted instances (both true positives and true negatives) to the total number of instances. While accuracy is a straightforward and widely used metric, it can be misleading in the case of imbalanced datasets where one class significantly outnumbers the other [28,29].

(15) $$Accuracy=\frac{True\;positives+True\;Negatives\;\;}{Total\;test\;samples\;}$$

The AUC is a performance measurement for classification problems at various threshold settings. The ROC curve is a plot of the true positive rate (TPR) against the false positive rate (FPR). The AUC represents the degree or measure of separability; it tells how much the model is capable of distinguishing between classes. An AUC of 1 indicates a perfect model, while an AUC of 0.5 suggests no discriminative power [30,31].

We did not use a dedicated test dataset to evaluate our model. Instead, we employed five-fold cross-validation, a widely recognized technique in EMG signal processing and machine learning analysis, to test our model. In this approach, the dataset is partitioned into five equally sized folds. During each iteration, one fold is used as the test set while the remaining four folds are combined to form the training set. This process is repeated five times, with each fold serving as the test set exactly once. By averaging the performance metrics across these five iterations, we obtain a robust measure of the model’s generalizability and performance. This technique ensures that every data point is used for both training and testing, providing a comprehensive and reliable evaluation of the model [32,33].

## 3. Results

This section presents the cross-validated performance scores of the four different models. The evaluation metrics include precision, recall, F1-score, accuracy, and AUC for the seven-class hand gesture test data. The results provide a comprehensive overview of each model’s ability to accurately classify the gestures, considering both overall performance and per-class performance. Table 2 shows the percentage scores of the five metrics for the linear regression model, while Table 3 and Table 4 present the scores for the SVM with linear and RBF kernels, respectively.

Table 5 and Table 6 display the scores for the Random Forest and kNN classifiers, respectively. Table 7 presents the overall scores for the four models, where SVM-1 and SVM-2 refer to the two versions of the SVM models using linear and RBF kernel functions, respectively.

## 4. Discussion

The primary objective of this research was to identify the most effective multi-class machine learning classifier for recognizing hand gestures using surface EMG signals obtained from the Myo armband module. The Myo armband is a user-friendly wearable device that captures EMG signals from eight specific locations on the subject’s arm. We designed an EMG data acquisition setup and selected seven distinct hand gestures for this study, collecting a total of 6822 hand gestures across these classes. Although we aimed to balance the dataset, classes 6 and 7 remained slightly unbalanced compared to the other classes. Through experimentation, we identified ten key features from the EMG segments to construct a feature vector for training four distinct ML models. We employed five-fold cross-validation to validate our model scores and calculated precision, recall, F1-score, accuracy, and AUC for each class, as well as the overall scores for each model.

The results demonstrated that the Random Forest classifier achieved over 91% across all metrics for all classes, consistently exceeding 95% in overall scores. Specifically, the precision, recall, F1-score, accuracy, and AUC scores for the Random Forest model were 95.58%, 96.60%, 95.57%, 95.38%, and 99.72%, respectively. This highlights the model’s robustness and reliability in gesture recognition tasks. Interestingly, the SVM model with an RBF kernel outperformed its linear kernel counterpart, underscoring the importance of kernel choice in SVM performance. The kNN model performed slightly below the Random Forest model. Conversely, the logistic regression model exhibited the lowest effectiveness among the classifiers evaluated.

The EMG data utilized in this project were obtained from a single subject, suggesting that even higher accuracy might be achievable with a larger and more diverse dataset. Addressing the unbalanced class distribution could further enhance model performance. However, inter-subject variability in EMG characteristics poses a challenge for generalizing across different individuals due to several factors. Physiological differences, such as variations in muscle structure, skin thickness, and the placement of muscles and nerves, significantly affect the EMG signals collected from different individuals. Signal variability is another issue, as EMG signals are inherently noisy and can be influenced by electrode placement, skin impedance, and movement artifacts, which can differ from person to person. Additionally, each individual may adapt and perform gestures slightly differently, leading to differences in EMG signal patterns even for the same gesture. Muscle fatigue also plays a role, as it can alter EMG signal characteristics, with the rate and manner of fatigue varying among individuals. These factors make it challenging to create a model that generalizes well across different subjects without significant customization or adaptation. Therefore, while a model trained on data from one individual may perform well for that individual, its performance may degrade when applied to a different person without additional training or calibration. This is why inter-subject variability is a key consideration in EMG-based gesture recognition studies.

As indicated by previous research, machine learning classifiers for bio-signals like EMG, EOG, and EEG are generally more suitable for intra-subject cases (within the same individual) rather than inter-subject cases (across different individuals) for several reasons. EMG signals vary significantly between different individuals due to factors such as muscle physiology, skin impedance, electrode placement, and unique movement patterns. This variability makes it challenging to develop a generalized model that performs well across multiple subjects. Moreover, EMG signals from the same individual tend to be more consistent over time, which allows for more reliable and stable model performance. Collecting sufficient and representative training data from multiple subjects to build a robust inter-subject model is often more difficult and resource-intensive compared to collecting data from a single subject. Intra-subject models can be fine-tuned to the specific signal characteristics of an individual, resulting in better performance and higher accuracy. This personalization helps in capturing the unique features of an individual’s EMG signals. Therefore, in this research, we employed intra-subject EMG classifiers to achieve higher accuracy and reliability.

A notable limitation of this study is the restricted number of hand gestures analyzed; only seven gestures were included. Future work will aim to incorporate a broader range of hand gestures, including those that are distinctly different from the current set.

Recognizing hand gestures has the potential to revolutionize various fields such as prosthetics for amputees, robotics, advanced human–computer interaction, rehabilitation, and assistive technologies for individuals with motor impairments. The advancements in this area could lead to significant improvements in the quality of life and functional independence for many individuals.

## 5. Conclusions

This study focused on identifying the optimal ML classifier for recognizing hand gestures using EMG signals from the Myo armband module. Among the models evaluated—Random Forest, SVM with linear and RBF kernels, logistic regression, and k-Nearest Neighbors—the Random Forest classifier demonstrated robust performance with mean scores consistently exceeding 95% across precision, recall, F1-score, accuracy, and AUC metrics. The SVM model with an RBF kernel outperformed its linear counterpart, highlighting kernel choice’s crucial role in classification accuracy. Conversely, logistic regression showed the least effectiveness. Challenges like inter-subject variability in EMG characteristics complicate model generalization across individuals, emphasizing the need for customized approaches. Future research should expand datasets, address class imbalance, and explore broader gesture sets to advance EMG-based gesture recognition for applications in prosthetics, robotics, and rehabilitation.

## Figures and Tables

**Table 1:** Hand gestures and their respective counts.

Activity/ Class ID	Finger/Gesture	Number of Segments (Counts)	Percentage (%)
1	Index finger extension	1039	15.23
2	Middle finger extension	1017	14.91
3	Ring finger extension	1045	15.32
4	Little finger extension	1084	15.89
5	Thumbs up	1016	14.89
6	Victory	726	10.64
7	Relaxed hand	895	13.12

**Table 2:** Cross-validated scores of the logistic regression model.

Metric/Class	Class 1	Class 2	Class 3	Class 4	Class 5	Class 6	Class 7
Precision	75.41	85.99	95.94	85.82	82.0	96.47	96.23
Recall	79.21	83.28	95.98	85.97	78.83	97.93	96.64
F1-score	77.24	84.60	95.94	85.89	80.36	97.19	96.43
Accuracy	79.21	83.28	95.98	85.97	78.83	97.93	96.64
AUC	96.06	97.67	99.73	98.18	96.74	99.99	99.90

**Table 3:** Cross-validated scores of the SVM model with a linear kernel.

Metric/Class	Class 1	Class 2	Class 3	Class 4	Class 5	Class 6	Class 7
Precision	75.03	87.10	95.67	88.64	87.86	98.07	96.61
Recall	84.21	85.54	96.45	88.28	78.83	98.21	95.30
F1-score	79.33	86.30	96.05	88.44	83.09	98.13	95.95
Accuracy	84.21	85.54	96.45	88.28	78.83	98.21	95.30
AUC	94.13	95.98	99.48	96.31	95.17	99.96	99.66

**Table 4:** Cross-validated scores of the SVM model with an ‘rbf’ kernel.

Metric/Class	Class 1	Class 2	Class 3	Class 4	Class 5	Class 6	Class 7
Precision	83.90	90.66	96.77	90.60	90.51	96.87	98.20
Recall	89.41	87.80	97.51	93.17	83.56	97.52	97.87
F1-score	86.54	89.20	97.13	91.86	86.89	97.19	98.03
Accuracy	89.41	87.80	97.51	93.17	83.53	97.52	97.87
AUC	97.14	97.57	99.63	98.48	96.57	99.93	99.78

**Table 5:** Cross-validated scores of the Random Forest classifier.

Metric/Class	Class 1	Class 2	Class 3	Class 4	Class 5	Class 6	Class 7
Precision	91.60	93.70	98.06	94.41	95.59	98.37	91.33
Recall	93.84	92.91	97.03	96.12	91.63	100	97.65
F1-score	92.68	93.27	97.54	95.24	93.56	99.18	97.49
Accuracy	93.84	92.91	97.03	96.12	91.63	100	97.65
AUC	99.50	99.55	99.82	99.73	99.53	99.99	99.94

**Table 6:** Cross-validated scores of the kNN classifier.

Metric/Class	Class 1	Class 2	Class 3	Class 4	Class 5	Class 6	Class 7
Precision	88.61	90.87	97.70	94.42	94.71	98.23	98.86
Recall	93.45	90.55	97.70	94.92	89.76	97.31	96.87
F1-score	90.96	90.86	97.70	94.66	92.16	98.76	97.85
Accuracy	93.45	90.85	97.70	94.92	89.79	99.31	96.87
AUC	98.66	98.55	99.63	99.37	98.14	99.96	99.85

**Table 7:** Cross-validated mean scores of the four models.

Metric/Model	LR	SVM-1	SVM-2	RF	kNN
Precision	88.27	89.86	92.5	95.58	94.77
Recall	88.26	89.51	92.41	95.6	94.69
F1-score	88.24	89.61	92.41	95.57	94.71
Accuracy	87.68	89.07	92.11	95.38	94.47
AUC	98.37	97.24	98.44	99.72	99.17

## Data Availability

Raw data used in this study are available upon request to the corresponding author.
